# Evaluation of the effectiveness and costs of inhaled methoxyflurane versus usual analgesia for prehospital injury and trauma: non-randomised clinical study

**DOI:** 10.1186/s12873-022-00664-y

**Published:** 2022-07-07

**Authors:** Murray D. Smith, Elise Rowan, Robert Spaight, Aloysius N. Siriwardena

**Affiliations:** 1 Community and Health Research Unit, University of Lincoln, Lincoln, UK; 2 Clinical Audit and Research Unit, East Midlands Ambulance Service NHS Trust, Nottingham, UK

**Keywords:** Prehospital, Emergency medical services, Analgesia, Pain, Ambulance, Methoxyflurane

## Abstract

**Background:**

We aimed to investigate clinical benefits and economic costs of inhaled methoxyflurane when used by ambulance staff for prehospital emergency patients with trauma. Comparison is to usual analgesic practice (UAP) in the UK in which patient records were selected if treatment had been with Entonox® or intravenous morphine or intravenous paracetamol.

**Methods:**

Over a 12-month evaluation period, verbal numerical pain scores (VNPS) were gathered from adults with moderate to severe trauma pain attended by ambulance staff trained in administering and supplied with methoxyflurane. Control VNPS were obtained from ambulance database records of UAP in similar patients for the same period. Statistical modelling enabled comparisons of methoxyflurane to UAP, where we employed an Ordered Probit panel regression model for pain, linked by observational rules to VNPS.

**Results:**

Overall, 96 trained paramedics and technicians from the East Midlands Ambulance Service NHS Trust (EMAS) prepared 510 doses of methoxyflurane for administration to a total of 483 patients. Comparison data extracted from the EMAS database of UAP episodes involved: 753 patients using Entonox®, 802 patients using intravenous morphine, and 278 patients using intravenous paracetamol. Modelling results included demonstration of faster pain relief with inhaled methoxyflurane (all p-values < 0.001). Methoxyflurane’s time to achieve maximum pain relief was estimated to be significantly shorter: 26.4 min (95%CI 25.0–27.8) versus Entonox® 44.4 min (95%CI 39.5–49.3); 26.5 min (95%CI 25.0–27.9) versus intravenous morphine 41.8 min (95%CI 38.9–44.7); 26.5 min (95%CI 25.1–28.0) versus intravenous paracetamol 40.8 (95%CI 34.7–46.9). Scenario analyses showed that durations spent in severe pain were significantly less for methoxyflurane. Costing scenarios showed the added benefits of methoxyflurane were achieved at higher cost, eg versus Entonox® the additional cost per treated patient was estimated to be £12.30.

**Conclusion:**

When administered to adults with moderate or severe pain due to trauma inhaled methoxyflurane reduced pain more rapidly and to a greater extent than Entonox® and parenteral analgesics. Inclusion of inhaled methoxyflurane to the suite of prehospital analgesics provides a clinically useful addition, but one that is costlier per treated patient.

**Supplementary Information:**

The online version contains supplementary material available at 10.1186/s12873-022-00664-y.

## Background

Acute pain is present in over 70% of those attended by Emergency Medical (ambulance) Services [1] for traumatic injury but is often inadequately treated [2]. Injury is the most common cause of moderate to severe pain in the prehospital setting [3], but in one UK study only one-fifth of patients with suspected fracture were administered opiates by ambulance staff and only a third given Entonox® [4].

Standard care, termed hereafter usual analgesic practice (UAP), for prehospital trauma pain includes non-drug measures (eg explanation, splinting) and a balanced analgesic approach depending on the cause of the pain: UAP for moderate to severe pain due to trauma includes morphine (intravenous or oral), paracetamol (intravenous) and Entonox® (an inhalational analgesic mixture of nitrous oxide and oxygen widely used prehospitally in the UK) [5]. Other agents, generally used in a stepwise fashion include non-opioids ± adjunct for mild pain (eg paracetamol or non-steroidal anti-inflammatory, usually ibuprofen) and weak opioids for moderate pain (eg codeine, dihydrocodeine alone or in combinations with paracetamol including co-codamol, co-dydramol) [5].

Barriers to adequate pain relief in the prehospital setting include poor pain assessment, limited analgesic choice or delivery routes and contraindications to effective drugs [2, 6]. Morphine, for example, requires intravenous cannulation and a paramedic to administer; potential side effects may restrict its use (sedation, respiratory depression, nausea, and hypotension). Solutions may include expanding the range of analgesics available, including to non-paramedic staff [6, 7].

Methoxyflurane (Penthrox®; Galen Ltd), a volatile fluorinated hydrocarbon anaesthetic, has analgesic properties in sub-anaesthetic doses [8]. Although easy to administer via a hand-held whistle-shaped inhaler, with a good safety profile [9, 10] and widely used as an inhalational analgesic in adults [11] and children for over 40 years in Australia and New Zealand [12, 13], there is limited evidence of clinical and cost effectiveness of methoxyflurane in the prehospital setting [9]. The European licence for methoxyflurane is for emergency relief of moderate to severe pain in conscious adult patients with trauma and associated pain [14]. It permits administration by both paramedics and emergency medical technicians (EMTs). Widespread roll out of methoxyflurane could thus be important in the UK where EMTs have been limited to administering Entonox® for moderate to severe acute pain.

A number of recent open-label randomised controlled trials (RCTs) conducted in Emergency Departments (EDs) have found that methoxyflurane relieves pain more quickly or more effectively or both compared with usual analgesia [15]. In the prehospital setting evidence for methoxyflurane is largely observational [9], based on indirect comparisons with Entonox®, or comparison with placebo in the ED [16]. An international retrospective chart review, comparing treatment in Australia with treatment in Europe, suggested that patients who received methoxyflurane experienced a shorter mean time to initial pain treatment during emergency care [17].

A rapid review suggested both methoxyflurane and Entonox® are safe and superior to placebo for reduction of moderate pain in the ED but found no evidence that either agent was better compared with each other [18]. A recent systematic review, indirectly comparing methoxyflurane with Entonox®, reported superior (but non-significant) pain relief at 15 min for methoxyflurane [19]. A previous observational study suggested that prehospital methoxyflurane was inferior to morphine in reducing pain score [20].

In this study, we aimed to evaluate the benefits and economic cost of adding methoxyflurane to prehospital care for adults with pain from traumatic injury. In particular, we focussed on the speed and degree of pain relief from use of methoxyflurane and the addition to costs if it was introduced into UAP.

## Methods

All methods were carried out in accordance with relevant ethics guidelines and regulations.

### Study design and setting

Supplementation of UAP with methoxyflurane was implemented by the East Midlands Ambulance Service National Health Service Trust (EMAS), a large English regional ambulance service, over an evaluation period running from December 2018 to November 2019. When a patient was administered methoxyflurane their episode data plus additional paramedic-recorded data were acquired for analysis.

Control episode data were extracted from the EMAS database of episodes of UAP care carried out under standard protocols, covering the same period as the methoxyflurane evaluation. Three distinct groups were constructed depending on the record of treatment: Entonox®, intravenous morphine (Morphine IV), intravenous paracetamol (Paracetamol IV). In addition, inclusion into each group required the patient match methoxyflurane’s indication on age, trauma, level of consciousness and severity of initial pain. Furthermore, for an episode to be included at least two pain scores must have been recorded with the first taken on or before the time of analgesic administration and the remainder taken during its period of effect.

We used a non-randomised, model-driven design to compare patients administered methoxyflurane with those treated with Entonox®, Morphine IV, Paracetamol IV, and, in each case, a prespecified hypothesis for test that methoxyflurane relieved pain faster than its comparator.

### History of study

Originally devised in November 2017 as a cluster randomised clinical trial (“MAPIT protocol version 1.0 221117.pdf”) and registered on 10/11/2017 ISRCTN #24016440 [21], approval to conduct the trial was refused by NHS Research Ethics in February 2018 [22] because there was insufficient resource to gain informed consent for purposes of research from patient participants. The trial was abandoned, the notification of which was added to its ISRCTN entry in November 2019.

In the meantime, methoxyflurane, being licensed for use by ambulance clinicians, was evaluated by EMAS from December 2018 to November 2019, the first NHS ambulance trust to do so. With EMAS making available to us their evaluation data plus UAP data obtained from their database of episodes of care, we designed, in December 2019, a retrospective observational database study (“MAPIT protocol version 1.3 031219.pdf”) that no longer required informed consent for the purpose of research from patient participants. Ethics approval for our retrospective observational database study was received from University of Lincoln Human Ethics Committee in December 2019 (“UoL Ethics_Letter091219.pdf”). In this paper we report on both the EMAS evaluation as well as our conduct of the approved retrospective observational database study.

### Staff training

Staff from four EMAS ambulance stations were recruited to volunteer to take part in training to administer methoxyflurane. Training sessions lasting one hour took place between October and December 2018. The first half of the session provided information about the drug, how it works, when to use it, who to use it on and contraindications. The second half was practical, using drug demonstration packs to show how to prepare the methoxyflurane delivery “whistle” and administer the drug to a patient. Trained staff received a laminated risk minimisation checklist (CHECK ALLL, see Table 1) and a leaflet with the data recording requirement, contact details for reporting an administration of the drug and key contraindications and safety reporting information.

**Table 1:**
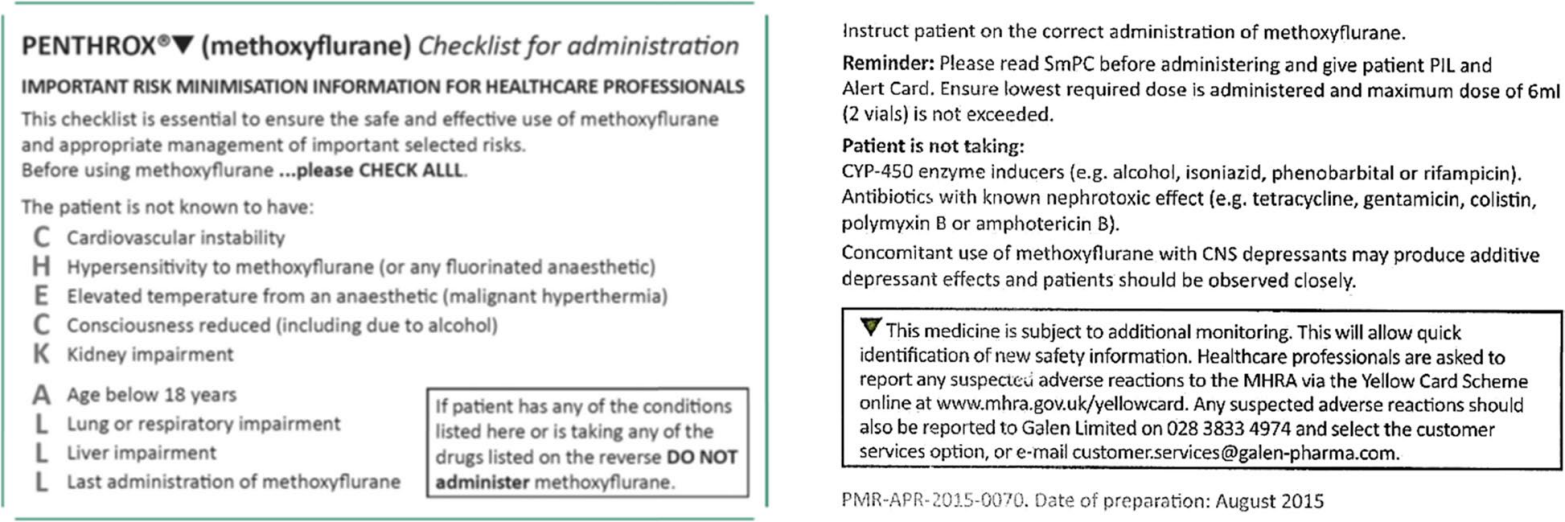
Risk minimisation (CHECK ALLL) checklist

Methoxyflurane was distributed to participating ambulance stations from a central store according to the Royal Pharmaceutical Society guidelines on The Safe and Secure Handling of Medicines and the EMAS Medicines Management Policy and stored in a locked drug cupboard in a locked room accessible by ambulance staff only. At the beginning of their shift, ambulance staff collected up to four single doses of methoxyflurane, each completing and signing a log recording the date and time the drug was collected, batch number and expiry date. Any drug used during the shift was reported on the log at the end of the shift and unused doses were returned and signed into the drug cupboard. It was emphasised that methoxyflurane should only be used as part of the evaluation.

### Patient recruitment

Adult (≥ 18 years) patients with moderate to severe traumatic pain from suspected single or multiple fracture, other injuries, and/or burns were eligible for recruitment if able to give verbal consent to treatment prior to methoxyflurane administration. Patients were ineligible if they had a medical contraindication to inhaled methoxyflurane, or lacked mental incapacity precluding informed consent. If it was deemed clinically appropriate or methoxyflurane was not effective, patients could be offered additional analgesia according to the scope of practice of the treating ambulance clinician. At conclusion, ambulance staff fitted patients with wristbands to enable ED staff to identify those patients that had received prehospital methoxyflurane and then at handover ED staff were issued with an information leaflet informing them that the patient had received methoxyflurane and what dose had been administered.

### Outcome measures

Patients given methoxyflurane were asked to score their pain prior to administration, 5 min later, and at 30 min or on arrival at ED, whichever was sooner. A verbal numerical pain score (VNPS) was used according to a severity-increasing 0–10 integer scale used routinely in UAP. We also used a 4-point categorisation of pain: severe (VNPS 7–10), moderate (VNPS 4–6), mild (VNPS 1–3), none (VNPS 0).

### Data analyses

Descriptive statistics were used to summarise patient demographics, treatments administered and pain scores. Analgesic performance against pain was assessed using an Ordered Probit panel regression model for pain, linked by observational rules to VNPS. Hypothetical scenario analyses were used to compare durations under analgesia spent in severe pain and to compare treatment costs. Computational work was undertaken using STATA®, Mathematica®, mathStatica® and MS-Excel®. A complete description of the development of the statistical model, hypothesis tests and scenario analyses is available in the electronic supplement.

### Cost analyses

From the perspective of the ambulance service, we contrasted the per patient cost of managing trauma pain with methoxyflurane against that of Entonox® and a weighted mix of parenteral morphine and paracetamol. The cost schedule upon which estimates are based is given in Table 2. Any other pain management costs that may be incurred but are not listed in the schedule are assumed to net to zero.

**Table 2:** Cost Schedule

Item	Unit	£	Source
***Methoxyflurane***			
Methoxyflurane	One vial contains 3 mL dose for vaporisation in a Penthrox® inhaler (Galen Ltd)	17.89	BNF
***Entonox®***			
Cylinder rental	Per month per ED-sized cylinder	4.84	EMAS
Cylinder exchange and refill	Per ED-sized cylinder	6.93	EMAS
Filter and mouthpiece	Clear-Guard Midi low volume breathing filter and mouthpiece (Intersurgical Ltd)	0.71	EMAS
***Morphine IV***			
Morphine sulfate	One ampoule contains 1 mL of solution 10 mg/1 mL for injection	1.145	Drug Tariff. Part VIIIA category A
***Paracetamol IV***			
Giving set	One single use set	0.43	EMAS
Paracetamol	One vial contains 100 mL of solution 1 g/100 mL for infusion (Accord Healthcare Ltd)	1.20	Drug Tariff. Part VIIIA category C
***Others***			
Cannula	One standard 18G (green) cannula	0.53	EMAS
Cannula pack	One standard cannula pack	0.70	EMAS
Syringe	One 10 mL syringe	0.04	EMAS
Needle	One blunt drawing up needle	0.04	EMAS
Tourniquet	One tourniquet	0.09	EMAS
Sodium Chloride	One ampoule contains 10 mL of solution 0.9% for injection	0.324	Drug Tariff. Part VIIIA category A
Ambulance see and treat or refer	Per incident	209.38	Reference Costs

Source details:

BNF British National Formulary. https://bnf.nice.org.uk/drug/methoxyflurane.html [Accessed April 2020]

EMAS Information supplied by East Midlands Ambulance Service NHS Trust

Drug Tariff NHS Electronic Drug Tariff, October 2020, available at http://www.drugtariff.nhsbsa.nhs.uk/#/00791628-DD/DD00791615/Home

Reference Costs National Schedule of NHS Costs 2018–19 – Ambulance

We assumed the cost of methoxyflurane was the product of its price per dose by its rate of use, thus allowing for dose wastage and second dosing. Next, we assumed that when managing pain with Entonox® that the average trauma patient uses half of the contents of a size ED cylinder (containing 700 L compressed gas when full). For parenteral morphine and paracetamol, we assumed single ampoules of both drugs were used along with, respectively, 3 and 2 ampoules of sodium chloride for flushing. In constructing the parenteral casemix we assume: 40% Morphine IV, 20% Paracetamol IV, 40% combined use of Morphine and Paracetamol IV. Finally, requiring additional on-scene crew to administer stronger analgesics (eg EMT crew requiring paramedic backup) was assumed to occur at the rate of one in every 50 patients and costed at the 2018/2019 NHS reimbursement rate.

## Results

### Methoxyflurane preparations and administration

**Fig. 1:**
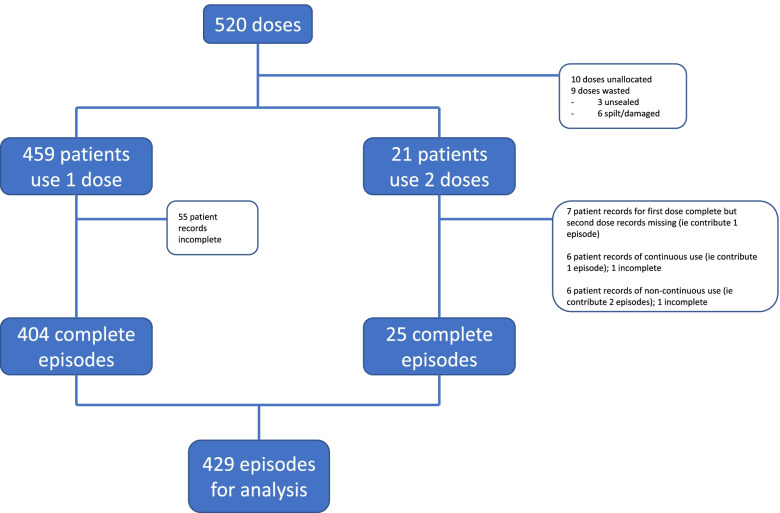
Methoxyflurane dose, patient and episode diagram

Overall, 96 clinicians were trained in administering methoxyflurane including 45 paramedics, 1 student paramedic and 50 EMTs. They prepared a total of 510 doses for administration to a grand total of 483 patients—10 doses from the overall supply of 520 were unallocated at the end of the evaluation period. A single dose was used by 458 patients, 21 patients used 2 doses (4.4%), and data on the usage or otherwise of one dose by 1 patient were completely missing. In total 9 doses (1.8%) were wasted comprising 3 doses which were unsealed, not used and discarded to waste, and 6 doses for which preparations resulted in failure at the first attempt at administration due to spillage or damage—these were replaced and a dose administered at the second attempt from available stock on hand. These details are depicted in Fig. 1.

Pain management was such that 217 of 479 patients used methoxyflurane and no other analgesic (45.3%). When it was used alongside other analgesics, methoxyflurane was first-line treatment for 89 patients and last in sequence for 142 patients. Clinicians prepared and administered most doses at the scene of the incident, yet 26 doses were prepared for administration during patient conveyance to hospital and 10 doses were initiated after arrival at hospital but before patient handover.

License violations for methoxyflurane were observed in age and condition. There were 4 patients aged under 18 years of whom 3 were administered methoxyflurane while the prepared dose was not administered but discarded to waste for the fourth. Two patients were ineligible according to the evaluation protocol because of Glasgow Coma Scale (GCS) scores of 13 or lower and 7 had their scores either misrecorded or missing altogether; only one of these 9 was not administered methoxyflurane, where their dose was discarded to waste after being prepared. Free-text record entries revealed that the patient in this case questioned whether the hospital ED would accept them as a patient if they were treated with methoxyflurane. At presentation, 10 patients were not trauma-classified yet 9 were subsequently administered methoxyflurane. Finally, methoxyflurane was administered to 2 patients that had given initial pain scores of 3 (mild) as well as to 5 of 6 patients missing their baseline pain score.

### Methoxyflurane compliance, discontinuation, and side effects

Ambulance crews used free-text entries to record patient compliance to inhalation instructions, to notify side effects and any discontinuations prior to completion of the dose. They recorded 13 patients (2.7%) nonadherent to instructions on use of the Penthrox® inhaler, 10 of whom were aged over 75 years. Despite their inhalation difficulties 6 patients persisted in trying to use their dose. Crews reported 36 occurrences of side-effects in 32 patients (6.7%) the severity of which forced 19 to discontinue methoxyflurane (4%). Table 3 lists the side-effects experienced by patients. Finally, one patient reported disliking methoxyflurane’s smell–described as being a “characteristic fruity odour” [14]—but we did not designate this to be a side-effect.

**Table 3:** Methoxyflurane side-effects by discontinuation in *n* = 32 patient reports

Side effect	Patient discontinued	Patient continued	Total
***Common or very common***			
Cough	6	4	10
Dizziness	1	-	1
Drowsiness	-	1	1
Dry mouth	1	-	1
Feeling abnormal	2	2	4
Headache	-	1	1
Mood altered	-	1	1
Nausea	4	1	5
Taste altered	7	2	9
***Uncommon***			
Anxiety	1	-	1
Oral discomfort	1	-	1
Paraesthesia	-	1	1
**Total**	23	13	36

### Comparison analgesic data

**Fig. 2:**
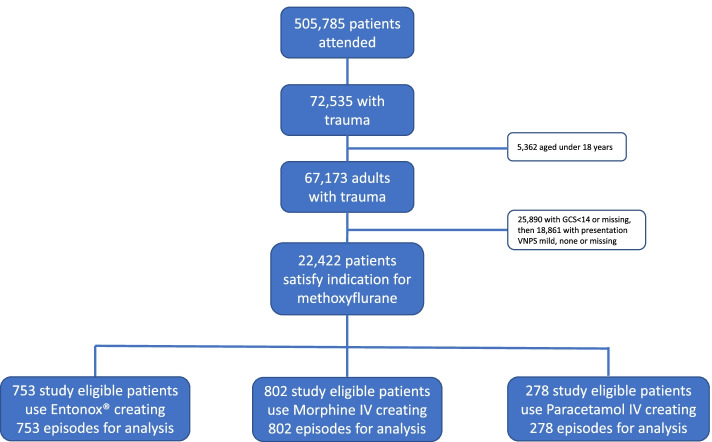
Comparator patient and episode diagram

From an EMAS supplied database of over 500,000 episodes of attended emergency care over the period December 2018 to November 2019, we identified 22,422 episodes that matched the licensed patient indication for treatment with methoxyflurane. This was further reduced to 1,833 episodes after imposing the study inclusion/exclusion restrictions. These were: (i) record of at least two VNPS scores, where the first had to be taken either on or before the time of analgesic administration and the remainder taken during its period of effect; and (ii) the only analgesic used was either Entonox® (753 patients), Morphine IV (802 patients) or Paracetamol IV (278 patients). These details are depicted in Fig. 2.

### Patient baseline characteristics

The patient sample in the methoxyflurane evaluation was mainly female (64.8%) with average age 63.1 years. Over 95% of patients recorded a GCS score of 15 indicating conscious and fully awake. Almost half of the patient sample presented with either a limb injury or fall with possibly secondary soft tissue injury too. Half as many again presented with either suspected neck of femur fracture or other hip injury with possible secondaries of limb injury or soft tissue injury. Table 4 lists these including descriptive statistics obtained for the comparison samples.

**Table 4:** Descriptive statistics by treatment group

Variable		Methoxyflurane (*n* = 483)	Entonox® (*n* = 753)	Morphine IV (*n* = 802)	Paracetamol IV (*n* = 278)
Sex	Female	64.8%	55.9%	58.6%	57.5%
	Male	35.2%	44.1%	41.4%	42.5%
Age	Mean (std dev; range)	63.1 (22.9; 16–98)	55.9 (22.1; 18–96)	61.4 (22.6; 18–99)	66.6 (20.3; 18–100)
GCS	14 or 15	98.1%	100%	100%	100%
	13 or lower	0.4%	-	-	-
	Other	1.5%	-	-	-
Trauma					
#1 Limb injury or fall ± soft tissue injury		45.8%	23.4%	44.6%	36.9%
#2 Suspected neck of femur (trauma) or hip injury ± limb or soft tissue injury		22.4%	10.4%	11.8%	21.5%
#3 Head, neck or back injury ± soft tissue injury		17.2%	27.3%	27.0%	13.2%
#4 Multisystem or multiple trauma, assault, stab wound, gunshot injury, penetrating trauma		6.4%	27.0%	10.4%	15.4%
#5 Abdominal or chest injury ± soft tissue injury		3.1%	10.8%	1.7%	9.1%
#6 Soft tissue or thermal injury		3.1%	1.1%	4.5%	4.0%
#7 Other		2.1%	-	-	-

### Pain scores

In Table 5 we report descriptive statistics associated with two pain scores–the pre-treatment baseline score ${S}_{0}$ and the final score ${S}_{T}$—taken from the estimation samples of episodes used in modelling. The estimation sample comprises complete cases, characterised by episodes containing at least two pain scores and associated timings, where the first score must be taken at a time no later than when the target analgesic was administered and the final pain score must be subject to influence by the target analgesic. The methoxyflurane estimation sample consisted of 423 patients providing 429 complete cases, where administrations were assumed statistically independent for 6 of 21 double-dosed patients as their second dose was noncontinuous to their first. The comparator estimation samples were equivalent to their extracted samples by construction.

**Table 5:** Estimation sample baseline ($${S}_{0})$$ and final ($${S}_{T}$$) pain scores by treatment group

	**Methoxyflurane**		**Entonox®**		**Paracetamol IV**		**Morphine IV**	
**Pain score**	${S}_{0}$ (*n* = 429)	${S}_{T}$ (*n* = 429)	${S}_{0}$ (*n* = 753)	${S}_{T}$ (*n *= 753)	${S}_{0}$ (*n* = 278)	${S}_{T}$ (*n* = 278)	${S}_{0}$ (*n* = 802)	${S}_{T}$ (*n* = 802)
**Mean**	8.7	4.5	7.8	5.6	7.2	4.8	8.3	5.0
**Standard deviation**	1.6	2.8	1.8	2.5	1.8	2.3	1.6	2.6
***Severe***								
10	46.4%	5.1%	23.2%	8.1%	16.9%	3.2%	34.4%	5.2%
9	11.0%	4.0%	13.7%	4.8%	8.3%	1.8%	14.7%	4.6%
8	21.5%	9.3%	24.8%	13.0%	23.0%	8.6%	24.1%	10.7%
7	10.0%	7.0%	12.5%	12.6%	15.8%	10.1%	11.2%	10.6%
***Moderate***								
6	6.1%	9.8%	11.4%	14.2%	15.1%	16.9%	7.6%	11.5%
5	4.0%	17.7%	8.9%	17.0%	16.6%	20.9%	6.2%	17.7%
4	0.7%	11.2%	5.4%	11.8%	4.3%	14.0%	1.8%	14.2%
***Mild***								
3	0.5%	8.9%	-	6.4%	-	8.6%	-	7.0%
2	-	10.7%	-	6.6%	-	7.6%	-	8.9%
1	-	4.9%	-	1.5%	-	3.2%	-	2.4%
***None***								
0	-	11.4%	-	4.0%	-	5.0%	-	7.2%
***Duration in minutes***								
First to baseline	8.4 (10.5; 0.0–85.5)		9.8 (10.3; 0.0–79.0)		17.1 (10.6; 0.4–52.5)		14.9 (10.6; 0.2–61.9)	
Baseline to final	26.1 (11.4; 1.0–47.5)		24.7 (16.1; 1.0–87.3)		26.7 (13.8; 5.0–68.5)		27.3 (16.7; 1.0–80.5)	

The baseline score ${S}_{0}$, assigned at time 0, was taken at time 0 for 92 methoxyflurane patients and, because missing for the remaining 331, was imputed, by protocol, as the score taken nearest beforehand to time 0 where that lead time averaged 8.4 min (median 5 min; std dev 10.5 min), although it did exceed 30 min for 17 patients. The average score at baseline lay within the range of severe pain for all treatment groups, with that classification applying to over 88% of all patients in the methoxyflurane group.

For methoxyflurane patients their final pain score ${S}_{T}$ was taken on average 26.1 min after administration (std dev 11.4 min; range 1–47.5 min) and averaged 4.5 (std dev 2.8) which is within the moderate range of pain. Final pain scores did decline in distribution to mild and none for over 35% of patients in the methoxyflurane group better than it did in the comparison samples: Entonox® 18.5%, Paracetamol IV 24.5%, Morphine IV 25.4%.

### Statistical modelling

Selected estimates for the three modelling comparisons are given in Table 6 (the complete table of estimation results is available in the electronic supplement). Interpretation is generally such that a positive/negative estimate implies an increase/decrease in pain as the variable increases/decreases in value, all other factors held constant. The variables are split into 4 main groups according to ineligibility, demographics, pathways and medicine use, and the column in which the parameter estimates appear indicates whether it pertains to methoxyflurane or comparator.

**Table 6:** Selected estimation results: main sample by comparison

	**Methoxyflurane**	**Entonox®**	**Methoxyflurane**	**Morphine IV**	**Methoxyflurane**	**Paracetamol IV**
**Estimation Sample**						
Patients	423	753	423	802	423	278
Observations	2907		3211		1934	
**Variable**						
***Ineligibilities***						
Under 18 (Yes = 1)	-0.064	-	-0.053	-	-0.075	-
Pain mild/none (Yes = 1)	-1.862**	-	-1.776**	-	-1.929**	-
GCS ineligible/missing (Yes = 1)	-0.779**	-	-0.740**	-	-0.796**	-
Trauma ineligible (Yes = 1)	0.284	-	0.271	-	0.312	-
***Demographics***						
Sex (F = 0, M = 1)	-0.402**	-0.285**	-0.387**	-0.048	-0.409**	-0.232
Age (years)	-0.003	-0.002	-0.003	-0.001	-0.003	-0.002
Trauma #1 (Yes = 1)	(reference)	(reference)	(reference)	(reference)	(reference)	(reference)
Trauma #2 (Yes = 1)	-0.152	0.222	-0.143	-0.157	-0.154	-0.228
Trauma #3 (Yes = 1)	-0.362*	0.019	-0.344*	-0.229	-0.359*	-0.136
Trauma #4 (Yes = 1)	-0.537**	-0.038	-0.502**	-0.187	-0.528**	-0.090
Trauma #5 (Yes = 1)	-0.048	0.062	-0.042	0.248	-0.041	-0.211
Trauma #6 (Yes = 1)	-0.650	-0.048	-0.622	-0.047	-0.658	0.181
***Pathways***						
Time, $t$	-0.071**		-0.108**		-0.081**	
Time squared, ${t}^{2}$	0.001**		0.001**		0.001**	
Methoxyflurane, $d$	0.730**	-	0.422	-	0.914**	-
Methoxyflurane x Time, $dt$	-0.135**	-	-0.088**	-	-0.128**	-
Methoxyflurane x Time^2^, $d{t}^{2}$	0.003**	-	0.002**	-	0.003**	-
***Medicine Use***						
Nonadherence (Yes = 1)	0.707*	-	0.674*	-	0.724*	-
Side-effect (Yes = 1)	0.064	-	0.069	-	0.085	-
Discontinue early (Yes = 1)	0.399	-	0.372	-	0.402	-
Influence Entonox® (Yes = 1)	-0.046	-	-0.019	-	-0.051	-
Influence potency 1 (Yes = 1)	0.003	-	-0.003	-	0.001	-
Influence potency 2 (Yes = 1)	-0.003	-	-0.006	-	-0.004	-
Influence potency 3 (Yes = 1)	0.253	-	0.237	-	0.270	-

^*^p < 0.05

^**^p < 0.01

The trauma numbering plan is given in Table 4

Sex differed significantly in the Morphine IV comparison (-0.387 vs -0.048; *p* = 0.016) but otherwise not in the other two comparisons (Entonox® -0.402 vs -0.283; *p* = 0.437, and Paracetamol IV -0.409 vs -0.232; *p* = 0.335). Males reported significantly less initial pain than females for those patients that were treated with methoxyflurane and for those patients that were treated with Entonox®. Age did not differ significantly across the three comparisons (*p* = 0.583, 0.487, 0.668 versus Entonox®, Morphine IV, Paracetamol IV, respectively) nor were any coefficients individually significant from zero. The joint test of trauma classifications showed no significant difference in coefficients in any comparison (*p* = 0.133, 0.494, 0.332 for versus Entonox®, Morphine IV, Paracetamol IV).

Within the methoxyflurane arm and relative to the reference group, people with limb injury or fall (possibly with soft tissue injury too), there were significant reductions in pain for people presenting with head, neck or back injury or multisystem trauma.

Pain was not significantly influenced by usage of concomitant analgesics in the methoxyflurane arm, joint tests yielding *p* = 0.899, 0.914, 0.877. However, there was a significant increase in pain when instructions on using the Penthrox® inhaler were not complied with. Occurrence of a side effect to methoxyflurane sufficient to cause its early discontinuation did not result in a significant increase in pain (*p* = 0.287, 0.289, 0.262).

Support for equality of effect was minimal (in each comparison *p* < 0.001 for the one-sided significance test), methoxyflurane relieved pain faster in each comparison. Estimates for duration to methoxyflurane’s trough pain averaged approximately 26.5 min (eg versus Entonox® the estimate was 26.41 min (standard deviation 0.73; 95%CI 24.98–27.83). All comparator trough pain estimates—Entonox® 44.44 (2.50; 95%CI 39.54–49.34), Morphine IV 41.76 (1.48; 95%CI 38.86–44.66), Paracetamol IV 40.75 (3.11; 95%CI 34.65–46.85) – were significantly larger.

### Pain pathways

In deriving the predicted pain pathways, parameter estimates were obtained using a per protocol sample in which methoxyflurane-ineligible patients were removed from the main sample (17 fewer patients resulting in 51 fewer pain scores). The predicted pain pathway by treatment for each comparison, set for the scenario to begin in severe pain, are given in Table 7 (the complete table of per protocol results is available in the electronic supplement).

**Table 7:** Scenario results: per protocol by comparator

Comparison	Predicted Pain Pathway	Duration in severe pain		Time to least pain		Level of least pain	
		**Time (mins)**	***p*-value**	**Time (mins)**	***p*-value**	**Pain**	***p*-value**
Methoxyflurane	$0.763+I\left\{t\ge 0.5\right\}[0.868\text{\hspace{0.05em}}-0.211t+0.004{t}^{2}]$	10.54 (se = 0.70)	-	26.94 (se = 0.74)	< 0.001	-1.97 (se = 0.18)	< 0.001
vs Entonox®	$0.763+I\left\{t\ge 0.5\right\}\left[0.799\text{\hspace{0.05em}}-0.072t+0.001{t}^{2}\right]$	^a^		44.96 (se = 2.49)		-0.82 (se = 0.18)	
Methoxyflurane	$0.441+I\left\{t\ge 0.5\right\}[0.540\text{\hspace{0.05em}}-0.200t+0.004{t}^{2}]$	10.47 (se = 0.69)	< 0.001	27.00 (se = 0.75)	< 0.001	-2.16 (se = 0.20)	0.010
vs Morphine IV	$0.441+I\left\{t\ge 1\right\}\left[0.550\text{\hspace{0.05em}}-0.111t+0.001{t}^{2}\right]$	20.09 (se = 1.12)		42.77 (se = 1.48)		-1.81 (se = 0.19)	
Methoxyflurane	$0.896+I\left\{t\ge 0.5\right\}[1.002\text{\hspace{0.05em}}-0.214t+0.004{t}^{2}]$	9.66 (se = 0.68)	< 0.001	27.07 (se = 0.76)	< 0.001	-1.89 (se = 0.28)	< 0.001
vs Paracetamol IV	$0.896+I\left\{t\ge 5\right\}\left[1.328\text{\hspace{0.05em}}-0.091t+0.001{t}^{2}\right]$	37.53 (se = 6.58)		45.77 (se = 3.11)		-0.76 (se = 0.29)	

^a^Not predicted to exit severe pain

**Fig. 3:**
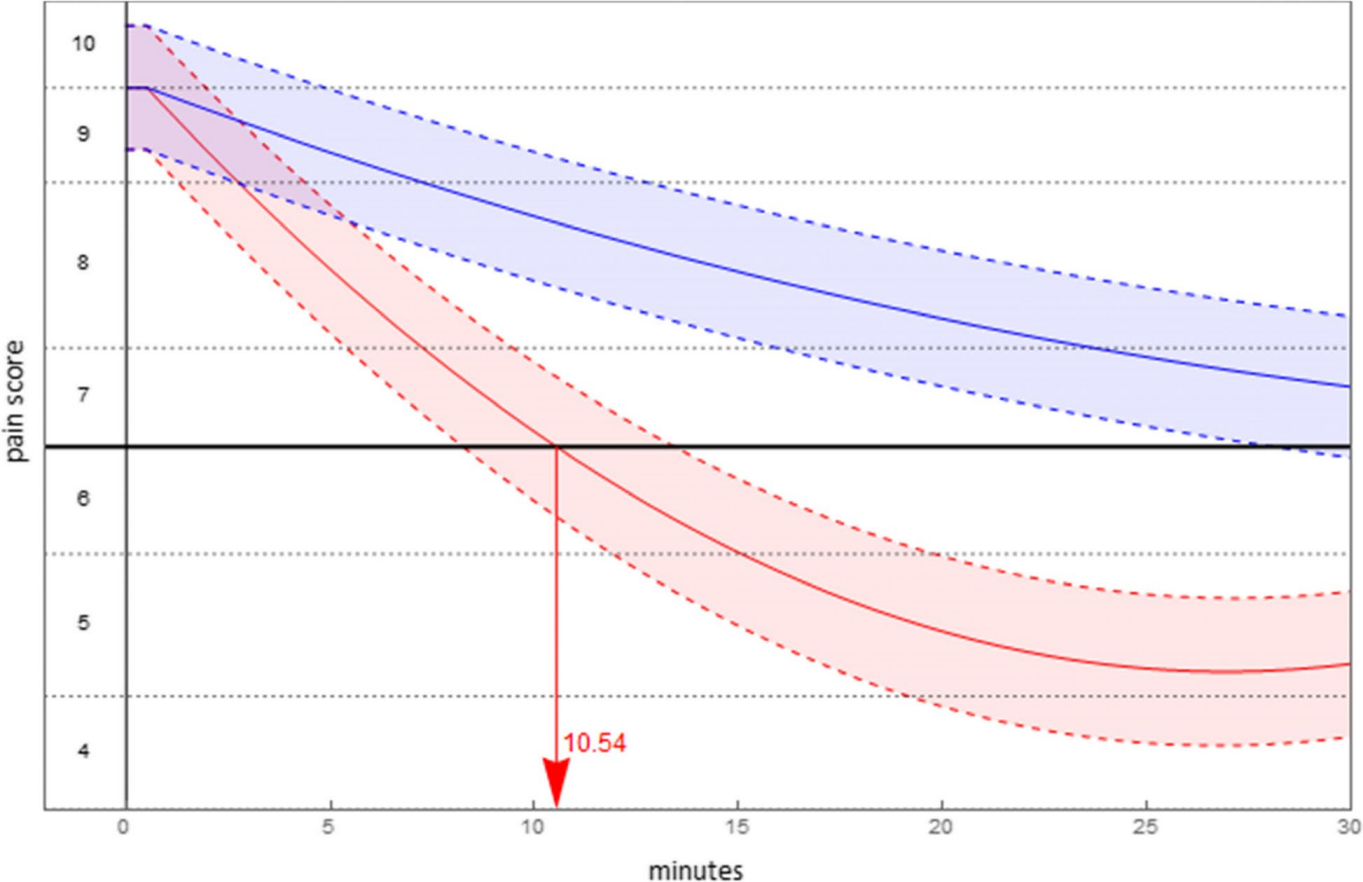
Predicted pain pathways (95% confidence interval shaded): methoxyflurane (red), Entonox® (blue)

**Fig. 4:**
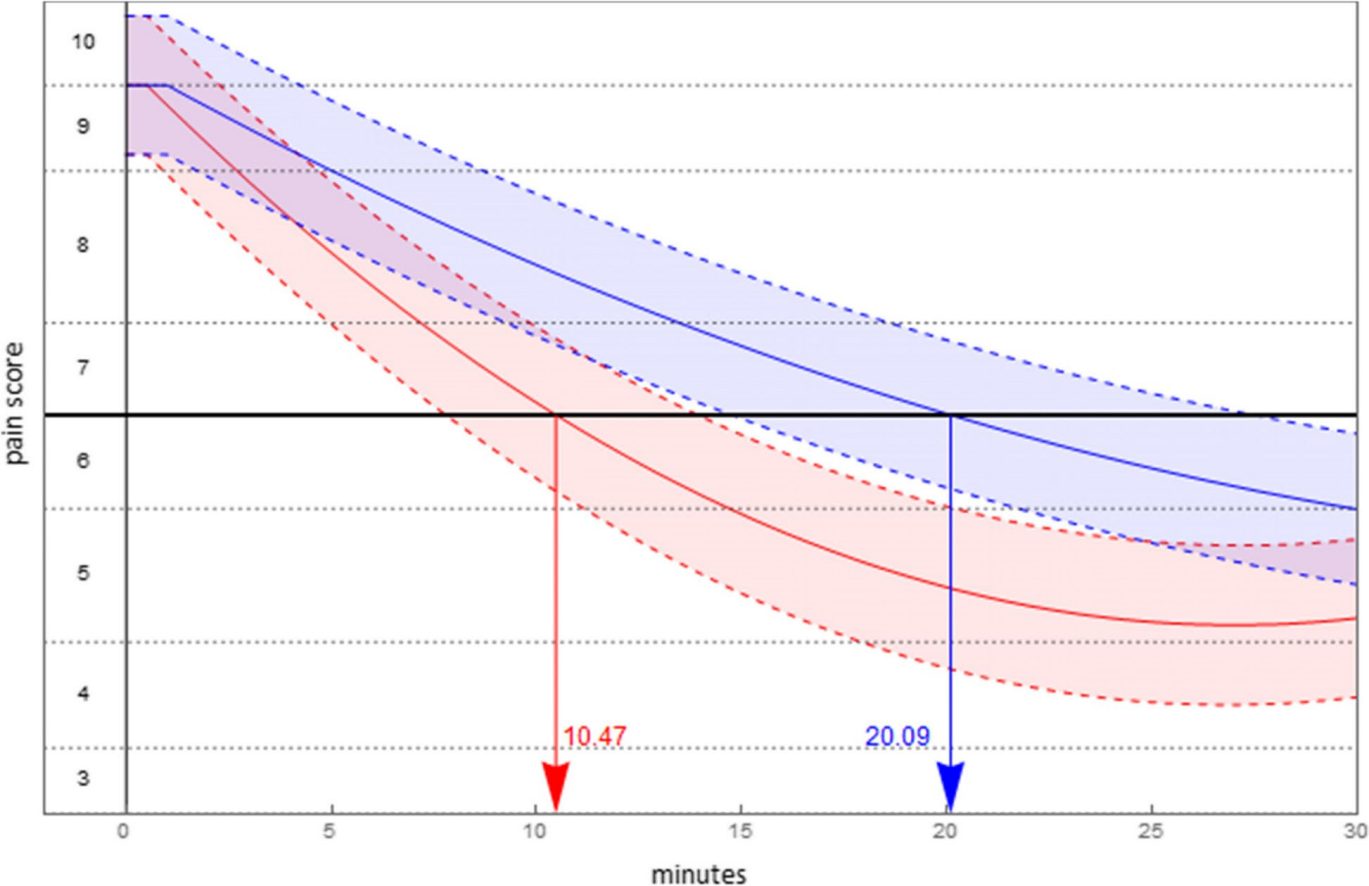
Predicted pain pathways (95% confidence interval shaded): methoxyflurane (red), Morphine IV (blue)

**Fig. 5:**
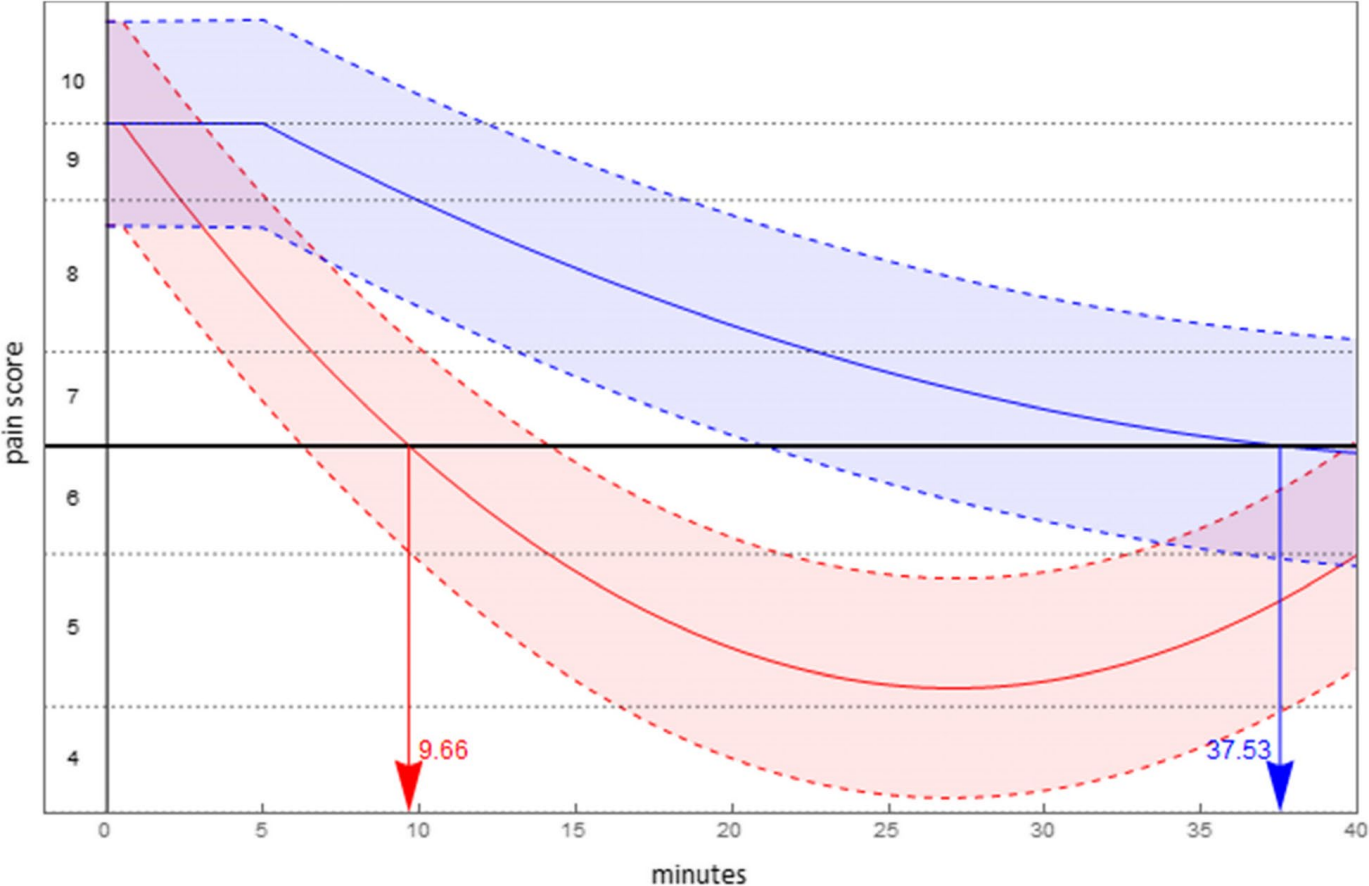
Predicted pain pathways (95% confidence interval shaded): methoxyflurane (red), Paracetamol IV (blue)

The duration of time until the level of pain was no longer classified severe was, for methoxyflurane, estimated to be a little over 10 min. Time to minimal pain (trough pain) is achieved at approximately 27 min for methoxyflurane. Figures 3, 4 and 5 display the predicted pain pathways for each comparison, with shading of 95% confidence intervals about those pathways and estimated durations in severe pain indicated. Also, for purposes of presentation the vertical axis has been transformed into pain score.

### Cost comparisons

We estimated the cost to the ambulance service of managing trauma pain with methoxyflurane to be £18.89 per patient, based on the overall usage rate observed in the evaluation (510 doses used by 483 patients). For Entonox®, added to breathing filter and mouthpiece we apportioned half, per assumption, the monthly bottle refill and rental charge to estimate the per patient cost as £6.60 (= £0.71 + (£4.84 + £6.93)/2). Managing trauma pain Morphine IV, we estimated the per patient cost to be £7.70, comprising single use of tourniquet, cannula and cannula pack, syringe, drawing up needle, 3 ampoules of sodium chloride and one ampoule of morphine sulfate per assumption; added too was provision for paramedic backup of £4.19 as per assumption (£209.38/50). Similarly, for Paracetamol IV we estimated the per patient cost to be £7.87. For the mix that combined use of Morphine IV and Paracetamol IV to manage trauma pain we estimated per patient cost to be £9.98. Finally, for the parenteral weighted casemix we estimated per patient cost to be £8.65.

Comparing ambulance service costs for the scenario that sees trauma pain managed with methoxyflurane as opposed to Entonox®, the added benefits due to use of methoxyflurane are estimated to incur additional cost per treated patient of £12.30. For the second scenario that compares methoxyflurane to a parenteral casemix, our estimate of the additional cost per treated patient was £10.24.

## Discussion

### Main findings

This is the largest pragmatic evaluation to date of prehospital methoxyflurane for adults with moderate or severe pain due to trauma. We evaluated the use, effect on pain and clinician reported adverse effects of methoxyflurane in a real-world prehospital ambulance setting, where the interventional drug was already licensed for use and where standard protocols and previous clinical experience and training meant that standard analgesic drugs could and would be used in conjunction with methoxyflurane. We found that methoxyflurane performed better than Entonox®, intravenous morphine or paracetamol in the extent and speed of pain reduction. The magnitude of reduction in pain was clinically important, particularly for those patients with initially severe pain and when compared to Entonox®. Methoxyflurane was the only drug administered for 45.3% of patients provided with this analgesic. Methoxyflurane was easy to administer in most patients and the rate of recorded adverse events was low.

The faster and greater reduction in pain judged against Entonox® was similar to effects observed in a previous study where methoxyflurane used in ED in one study was compared indirectly with Entonox® used prehospitally in another, where each included study used placebo as a comparator [19]. This study is limited by the indirect comparisons made and the different settings and outcomes used [19]. Entonox®, also poses problems of storage, maintenance, cylinder size affecting use in confined spaces, poorly fitting masks or mouthpieces, or difficulties for some patients of activating the demand valve [23].

The advantages of methoxyflurane in achieving faster pain reduction was consistent with findings from a number of recent open-label RCTs [15]. This includes an RCT comparing methoxyflurane with standard analgesia in patients with moderate to severe trauma pain conducted in emergency units in Italy which showed that methoxyflurane was more effective and quicker median time to onset of pain relief was also quicker (median onset 9 min vs 15 min) at reducing pain than standard analgesic treatment (intravenous paracetamol or ketoprofen for moderate pain and intravenous morphine for severe pain) for moderate or severe pain [24]. Another RCT conducted in EDs in Spain showed a greater mean decrease from baseline in pain score for methoxyflurane compared with standard analgesic treatment at all points up to 20 min [25].

A further randomised, double-blind, placebo-controlled trial in EDs in France, comparing methoxyflurane vs placebo, both combined with usual analgesia [26]. showed a median time to pain relief (≤ 30 mm on 100-mm VAS) of 35 min [95%CI 28–62] for methoxyflurane vs not reached in the placebo arm (hazard ratio 1.93, 95%CI 1.43–2.60, P < 0.001) and most effective in the severe pain subgroup. Finally, an RCT in a tertiary hospital ED in Australia in 120 adult patients with initial pain score of 8 or above randomised 1:1 to receive either inhaled methoxyflurane (3 mL) or standard analgesic treatment at ED found no significant difference in primary outcome (proportion of patients achieving at least a 50% drop in the pain score at 30 min) but a significantly higher proportion of patients in the methoxyflurane arm reported a greater than 2-point drop in pain score and lower median pain scores at all time points [27].

In the prehospital setting comparing methoxyflurane with intramuscular tramadol in a cluster randomised cross-over trial for people with moderate to severe pain due to musculoskeletal trauma in Singapore, methoxyflurane was superior to tramadol in efficacy, speed of onset and administration, but with more minor adverse effects [23].

Adverse effects were uncommonly reported in this study. Recent RCTs of methoxyflurane recorded adverse effects including headache, dizziness and sleepiness [15]. There are concerns that methoxyflurane may be potentially harmful to healthcare staff through passive inhalation (‘off-gassing’) but this was not borne out by a study in ED nurses in France [28] and has not been a problem in over four decades of prehospital use in Australia and New Zealand.

### Strengths and limitations

We were able to account for use of other drugs using carefully constructed statistical models, considering the most likely confounders including age and sex, in adults who had suffered traumatic injury. We accounted for the nature of the injury but there may have been other unknown confounders that led to the differences we found. Because ambulance clinicians were not blinded to assessment of outcomes it is possible that pain scores were affected by knowledge that methoxyflurane was being used. To mitigate this, the importance of accurate pain assessment based on pain scores reported by the patient was emphasised during training.

The cost analyses and comparisons were based on reference and local costs, but these were presented transparently so that they can be reviewed and revised considering differences or future changes in costs of methoxyflurane or the comparators.

## Conclusions

Our findings suggest that methoxyflurane provides an effective prehospital alternative to Entonox® and intravenous options for moderate to severe pain in patients with trauma and can be safely used by paramedics and EMTs. This study points to methoxyflurane acting earlier and with a greater reduction in pain than Entonox® or intravenous paracetamol and also acting more quickly with a similar reduction in pain compared with intravenous morphine.

The more rapid is analgesia may be particularly helpful in the prehospital environment where speed of stabilisation and transfer of patient is important, where cannulation may be difficult or where some ambulance personnel may not be trained to cannulate or administer intravenous analgesics. This will have implications for prehospital guidance in trauma in the UK and Europe which have been updated in the UK [5] to include methoxyflurane as an analgesic option for trauma pain in adults in May 2021.

Further research, using randomised controlled designs would be needed to confirm the relative benefits of methoxyflurane compared to intravenous morphine or paracetamol for pain due to trauma in adults in the prehospital setting. Other potential benefits, for example on speed of delivery to the ED and patient reported experience should also be investigated [29].

## Supplementary Information

**Additional file 1: Supplement 1.** Estimation sample and outputs for methoxyflurane against each comparator: main sample. 

**Additional file 2: Supplement 2.** Estimation sample and outputs for methoxyflurane against each comparator: per protocol license indication sample.

**Additional file 3: Supplement3.** Statistics.

## Data Availability

The data that support the findings of this study are available from EMAS but restrictions apply to the availability of these data which are not publicly available. Data are however available from the corresponding author upon reasonable request and with permission of EMAS.

## Abbreviations

BNF: British National Formulary
CI: Confidence interval
ED: Emergency Department
EMAS: East Midlands Ambulance Service National Health Service Trust
EMT: Emergency Medical Technician
GCS: Glasgow Coma Scale
NHS: National Health Service
RCT: Randomised controlled trial
se: Standard error
std dev: Standard deviation
VNPS: Verbal numerical pain scores
UAP: Usual analgesic practice
UK: United Kingdom
