# Supplementary material for: Evaluation of the effectiveness and costs of inhaled methoxyflurane versus usual analgesia for prehospital injury and trauma: non-randomised clinical study
Source: BMC Emerg Med. 2022 Jul 7;22:122. doi: 10.1186/s12873-022-00664-y (PMC9261021; doi:10.1186/s12873-022-00664-y)
Supplement: Supplementary file 1 — Additional file 1: Supplement 1. Estimation sample and outputs for methoxyflurane against each comparator: main sample. [file 12873_2022_664_MOESM1_ESM.docx]

Supplement 1: Estimation sample and outputs for methoxyflurane against each comparator

Main sample

Table S1.1 Patient numbers and pain scores

|  | **Methoxyflurane** | **Entonox®** | **Morphine IV** | **Paracetamol IV** |
| --- | --- | --- | --- | --- |
| **Patients**  Single dose $n1$  Double dose $n21$  Double dose $n22$  Double dose $n23$  **Total** | 404  7  6  6  423 | -  -  -  -  753 | -  -  -  -  802 | -  -  -  -  278 |
| **Patient pain scores**  2 recorded  3 recorded  4 recorded  5 recorded  6 recorded  7 recorded | 100%  79.2%  14.9%  4.5%  1.4%  0.2% | 100%  15.4%  1.9%  0.3%  -  - | 100%  32.9%  7.9%  1.4%  -  - | 100%  31.3%  5.8%  1.4%  0.7%  - |

Table S1.2 Model parameter estimates

| **Variable** | **Methoxyflurane** | **Entonox®** | | **Methoxyflurane** | **Morphine IV** | **Methoxyflurane** | | **Paracetamol IV** |
| --- | --- | --- | --- | --- | --- | --- | --- | --- |
| Time  $t$ | -0.071**  (0.004; 0.000) | | | -0.108**  (0.004; 0.000) | | -0.081**  (0.006; 0.000) | | |
| Time squared  $t^{2}$ | 0.001**  (0.000; 0.000) | | | 0.001**  (0.000; 0.000) | | 0.001**  (0.000; 0.000) | | |
| Treatment  $d$ | 0.730**  (0.251; 0.004) | | - | 0.422  (0.259; 0.104) | - | 0.914**  (0.327; 0.005) | - | |
| Treatment x Time  $dt$ | -0.135**  (0.012; 0.000) | | - | -0.088**  (0.011; 0.000) | - | -0.128**  (0.013; 0.000) | - | |
| Treatment x Time squared  $dt^{2}$ | 0.003**  (0.000; 0.000) | | - | 0.002**  (0.000; 0.000) | - | 0.003**  (0.000; 0.000) | - | |
| Sex | -0.402**  (0.122; 0.001) | -0.285**  (0.088; 0.001) | | -0.387**  (0.117; 0.001) | -0.048  (0.079; 0.544) | -0.409**  (0.124; 0.001) | | -0.232  (0.136; 0.087) |
| Age | -0.003  (0.003; 0.215) | -0.002  (0.002; 0.447) | | -0.003  (0.003; 0.226) | -0.001  (0.002; 0.710) | -0.003  (0.003; 0.208) | | -0.002  (0.003; 0.584) |
| Under 18 | -0.120  (0.313; 0.701) | - | | -0.105  (0.301; 0.727) | - | -0.133  (0.328; 0.685) | | - |
| No GCS | -0.779**  (0.216; 0.000) | - | | -0.740**  (0.206; 0.000) | - | -0.796**  (0.225; 0.000) | | - |
| No Trauma | 0.284  (0.328; 0.387) | - | | 0.271  (0.312; 0.385) | - | 0.312  (0.333; 0.350) | | - |
| Mild/No Pain | -1.862**  (0.450; 0.000) | - | | -1.776**  (0.428; 0.000) | - | -1.929**  (0.460; 0.000) | | - |
| Trauma #2 | -0.152  (0.148; 0.305) | 0.222  (0.147; 0.131) | | -0.143  (0.141; 0.313) | -0.157  (0.108; 0.145) | -0.154  (0.150; 0.305) | | -0.228  (0.231; 0.322) |
| Trauma #3 | -0.362*  (0.150; 0.016) | 0.019  (0.103; 0.852) | | -0.344*  (0.143; 0.016) | -0.229  (0.123; 0.063) | -0.359*  (0.153; 0.019) | | -0.136  (0.195; 0.487) |
| Trauma #4 | -0.537**  (0.196; 0.006) | -0.038  (0.159; 0.809) | | -0.502**  (0.187; 0.007) | -0.187  (0.112; 0.096) | -0.528**  (0.200; 0.008) | | -0.090  (0.182; 0.622) |
| Trauma #5 | -0.048  (0.313; 0.879) | 0.062  (0.155; 0.689) | | -0.042  (0.299; 0.887) | 0.248  (0.146; 0.090) | -0.041  (0.317; 0.898) | | -0.211  (0.222; 0.342) |
| Trauma #6 | -0.650  (0.390; 0.096) | -0.048  (0.251; 0.849) | | -0.622  (0.372; 0.094) | -0.047  (0.184; 0.800) | -0.658  (0.399; 0.099) | | 0.181  (0.465; 0.697) |
| Influence E | -0.046  (0.133; 0.732) | - | | -0.019  (0.126; 0.882) | - | -0.051  (0.133; 0.700) | | - |
| Influence P1 | 0.003  (0.162; 0.987) | - | | -0.003  (0.154; 0.986) | - | 0.001  (0.165; 0.993) | | - |
| Influence P2 | -0.003  (0.236; 0.988) | - | | -0.006  (0.227; 0.979) | - | -0.004  (0.242; 0.986) | | - |
| (continued next page) | | | | | | | | |
| Influence P3 | 0.253  (0.254; 0.319) | - | | 0.237  (0.242; 0.326) | - | 0.270  (0.257; 0.293) | | - |
| Compliance | 0.707*  (0.351; 0.044) | - | | 0.674*  (0.331; 0.042) | - | 0.724*  (0.349; 0.038) | | - |
| Side-effect | 0.064  (0.226; 0.776) | - | | 0.069  (0.215; 0.748) | - | 0.085  (0.230; 0.710) | | - |
| Discontinue | 0.399  (0.274; 0.145) | - | | 0.372  (0.259; 0.151) | - | 0.402  (0.273; 0.140) | | - |
| Random effect  $\sigma_{u}^{2}$ | 0.855**  (0.080; 0.000) | | | 0.698**  (0.067; 0.000) | | 0.820**  (0.090; 0.000) | | |
| Cut #1  $\alpha_{1}$ | -3.593**  (0.174; 0.000) | | | -3.713**  (0.192; 0.000) | | -3.570**  (0.285; 0.000) | | |
| Cut #2  $\alpha_{2}$ | -3.394**  (0.168; 0.000) | | | -3.526**  (0.188; 0.000) | | -3.308**  (0.278; 0.000) | | |
| Cut #3  $\alpha_{3}$ | -2.921**  (0.159; 0.000) | | | -3.072**  (0.183; 0.000) | | -2.842**  (0.266; 0.000) | | |
| Cut #4  $\alpha_{4}$ | -2.611**  (0.155; 0.000) | | | -2.750**  (0.180; 0.000) | | -2.493**  (0.260; 0.000) | | |
| Cut #5  $\alpha_{5}$ | -2.085**  (0.150; 0.000) | | | -2.241**  (0.178; 0.000) | | -1.977**  (0.256; 0.000) | | |
| Cut #6  $\alpha_{6}$ | -1.418**  (0.146; 0.000) | | | -1.598**  (0.173; 0.000) | | -1.223**  (0.254; 0.000) | | |
| Cut #7  $\alpha_{7}$ | -0.916**  (0.145; 0.000) | | | -1.145**  (0.172; 0.000) | | -0.692**  (0.253; 0.006) | | |
| Cut #8  $\alpha_{8}$ | -0.458**  (0.145; 0.002) | | | -0.705**  (0.171; 0.000) | | -0.234  (0.253; 0.354) | | |
| Cut #9  $\alpha_{9}$ | 0.321*  (0.145; 0.027) | | | 0.033  (0.171; 0.845) | | 0.524*  (0.252; 0.038) | | |
| Cut #10  $\alpha_{10}$ | 0.759**  (0.147; 0.000) | | | 0.440*  (0.171; 0.010) | | 0.890**  (0.256; 0.000) | | |

$$Notes: {\text{estimate} \atop\text{(std err; }\text{p}\text{-value)}}, *p<0.05, **p<0.01$$

Table S1.3 Estimation statistics

| **Statistic** | **Methoxyflurane** | **Entonox®** | **Methoxyflurane** | **Morphine IV** | **Methoxyflurane** | **Paracetamol IV** |
| --- | --- | --- | --- | --- | --- | --- |
| Total observations | 2907 | | 3211 | | 1934 | |
| Log-pseudolikelihood | -5789.347 | | -6341.488 | | -3810.583 | |
| ***Hypothesis tests*** |  |  |  |  |  |  |
| Clinical efficacy | -11.78 (p<0.001) |  | -7.98 (p<0.001) |  | -9.96 (p<0.001) |  |
| All traumas | 8.45 (p=0.133) |  | 4.39 (p=0.494) |  | 5.75 (p=0.332) |  |
| Other analgesics | 1.07 (p=0.899) |  | 0.97 (p=0.914) |  | 1.21 (p=0.877) |  |
| Serious adverse event | 2.50 (p=0.287) |  | 2.48 (p=0.289) |  | 2.68 (p=0.262) |  |
| ***Time to trough pain*** | 26.41  (0.73; 24.98-27.83) | 44.44  (2.50; 39.54-49.34) | 26.46  (0.74; 25.02-27.91) | 41.76  (1.48; 38.86-44.66) | 26.54  (0.75; 25.07-28.00) | 40.75  (3.11; 34.65-46.85) |
