# Supplementary material for: Evaluation of the effectiveness and costs of inhaled methoxyflurane versus usual analgesia for prehospital injury and trauma: non-randomised clinical study
Source: BMC Emerg Med. 2022 Jul 7;22:122. doi: 10.1186/s12873-022-00664-y (PMC9261021; doi:10.1186/s12873-022-00664-y)
Supplement: Supplementary file 2 — Additional file 2: Supplement 2. Estimation sample and outputs for methoxyflurane against each comparator: per protocol license indication sample. [file 12873_2022_664_MOESM2_ESM.docx]

Supplement 2: Estimation sample and outputs for methoxyflurane against each comparator

Per protocol license indication sample

Table S2.1 Patient numbers and pain scores

|  | **Methoxyflurane** | **Entonox®** | **Morphine IV** | **Paracetamol IV** |
| --- | --- | --- | --- | --- |
| **Patients**  Single dose $n1$  Double dose $n21$  Double dose $n22$  Double dose $n23$  **Total** | 387  7  6  6  406 | -  -  -  -  753 | -  -  -  -  802 | -  -  -  -  278 |
| **Patient pain scores**  2 recorded  3 recorded  4 recorded  5 recorded  6 recorded  7 recorded | 100%  79.6%  14.8%  4.7%  1.5%  0.2% | 100%  15.4%  1.9%  0.3%  -  - | 100%  32.9%  7.9%  1.4%  -  - | 100%  31.3%  5.8%  1.4%  0.7%  - |

Table S2.2 Model parameter estimates

| **Variable** | **Methoxyflurane** | **Entonox®** | | **Methoxyflurane** | **Morphine IV** | **Methoxyflurane** | | **Paracetamol IV** |
| --- | --- | --- | --- | --- | --- | --- | --- | --- |
| Time  $t$ | -0.071**  (0.004; 0.000) | | | -0.108**  (0.004; 0.000) | | -0.081**  (0.006; 0.000) | | |
| Time squared  $t^{2}$ | 0.001**  (0.000; 0.000) | | | 0.001**  (0.000; 0.000) | | 0.001**  (0.000; 0.000) | | |
| Treatment  $d$ | 0.742**  (0.258; 0.004) | | - | 0.431  (0.265; 0.104) | - | 0.929**  (0.333; 0.005) | - | |
| Treatment x Time  $dt$ | -0.136**  (0.012; 0.000) | | - | -0.088**  (0.011; 0.000) | - | -0.128**  (0.013; 0.000) | - | |
| Treatment x Time squared  $dt^{2}$ | 0.003**  (0.000; 0.000) | | - | 0.002**  (0.000; 0.000) | - | 0.003**  (0.000; 0.000) | - | |
| Sex | -0.389**  (0.126; 0.002) | -0.285**  (0.089; 0.001) | | -0.373**  (0.120; 0.002) | -0.048  (0.079; 0.542) | -0.396**  (0.128; 0.002) | | -0.233  (0.136; 0.087) |
| Age | -0.004  (0.003; 0.211) | -0.002  (0.002; 0.451) | | -0.003  (0.003; 0.223) | -0.001  (0.002; 0.710) | -0.004  (0.003; 0.205) | | -0.002  (0.003; 0.588) |
| Trauma #2 | -0.149  (0.151; 0.322) | 0.223  (0.147; 0.131) | | -0.141  (0.144; 0.329) | -0.157  (0.108; 0.146) | -0.152  (0.153; 0.320) | | -0.229  (0.232; 0.323) |
| Trauma #3 | -0.369*  (0.153; 0.016) | 0.019  (0.103; 0.854) | | -0.351*  (0.146; 0.016) | -0.229  (0.123; 0.063) | -0.366*  (0.156; 0.019) | | -0.135  (0.196; 0.490) |
| Trauma #4 | -0.540**  (0.197; 0.006) | -0.038  (0.160; 0.812) | | -0.503**  (0.188; 0.007) | -0.187  (0.112; 0.095) | -0.531**  (0.202; 0.008) | | -0.089  (0.183; 0.625) |
| Trauma #5 | -0.050  (0.317; 0.874) | 0.061  (0.156; 0.694) | | -0.045  (0.302; 0.883) | 0.248  (0.146; 0.090) | -0.043  (0.321; 0.893) | | -0.212  (0.223; 0.342) |
| Trauma #6 | -0.656  (0.392; 0.094) | -0.047  (0.252; 0.852) | | -0.627  (0.373; 0.092) | -0.047  (0.185; 0.797) | -0.666  (0.401; 0.097) | | 0.182  (0.467; 0.696) |
| Influence E | -0.050  (0.138; 0.717) | - | | -0.023  (0.131; 0.859) | - | -0.057  (0.139; 0.683) | | - |
| Influence 1 | 0.023  (0.165; 0.890) | - | | 0.016  (0.157; 0.918) | - | 0.020  (0.169; 0.904) | | - |
| Influence 2 | 0.006  (0.244; 0.980) | - | | 0.004  (0.235; 0.987) | - | -0.005  (0.251; 0.983) | | - |
| Influence 3 | 0.171  (0.271; 0.528) | - | | 0.157  (0.256; 0.541) | - | 0.186  (0.274; 0.496) | | - |
| Compliance | 0.693  (0.359; 0.054) | - | | 0.660  (0.338; 0.051) | - | 0.712*  (0.357; 0.046) | | - |
| Side-effect | 0.059  (0.240; 0.807) | - | | 0.065  (0.227; 0.774) | - | 0.082  (0.243; 0.737) | | - |
| Discontinue | 0.427  (0.299; 0.153) | - | | 0.397  (0.282; 0.160) | - | 0.427  (0.298; 0.152) | | - |
| (continued next page) | | | | | | | | |
| Random effect  $\sigma_{u}^{2}$ | 0.876**  (0.082; 0.000) | | | 0.712**  (0.068; 0.000) | | 0.849**  (0.093; 0.000) | | |
| Cut #1  $\alpha_{1}$ | -3.602**  (0.175; 0.000) | | | -3.718**  (0.193; 0.000) | | -3.585**  (0.287; 0.000) | | |
| Cut #2  $\alpha_{2}$ | -3.397**  (0.169; 0.000) | | | -3.527**  (0.188; 0.000) | | -3.313**  (0.279; 0.000) | | |
| Cut #3  $\alpha_{3}$ | -2.923**  (0.160; 0.000) | | | -3.072**  (0.184; 0.000) | | -2.846**  (0.267; 0.000) | | |
| Cut #4  $\alpha_{4}$ | -2.620**  (0.156; 0.000) | | | -2.756**  (0.180; 0.000) | | -2.506**  (0.261; 0.000) | | |
| Cut #5  $\alpha_{5}$ | -2.090**  (0.151; 0.000) | | | -2.243**  (0.178; 0.000) | | -1.983**  (0.257; 0.000) | | |
| Cut #6  $\alpha_{6}$ | -1.423**  (0.147; 0.000) | | | -1.602**  (0.174; 0.000) | | -1.227**  (0.255; 0.000) | | |
| Cut #7  $\alpha_{7}$ | -0.921**  (0.145; 0.000) | | | -1.149**  (0.172; 0.000) | | -0.694**  (0.254; 0.006) | | |
| Cut #8  $\alpha_{8}$ | -0.458**  (0.145; 0.002) | | | -0.705**  (0.171; 0.000) | | -0.230  (0.254; 0.364) | | |
| Cut #9  $\alpha_{9}$ | 0.317*  (0.146; 0.030) | | | 0.028  (0.171; 0.868) | | 0.520*  (0.253; 0.040) | | |
| Cut #10  $\alpha_{10}$ | 0.763**  (0.148; 0.000) | | | 0.441*  (0.171; 0.010) | | 0.896**  (0.257; 0.000) | | |

$$Notes: {\text{estimate} \atop\text{(std err; }\text{p}\text{-value)}}, *p<0.05, **p<0.01$$

Table S2.3 Estimation statistics

| **Statistic** | **Methoxyflurane** | **Entonox®** | **Methoxyflurane** | **Morphine IV** | **Methoxyflurane** | **Paracetamol IV** |
| --- | --- | --- | --- | --- | --- | --- |
| Total observations | 2856 | | 3160 | | 1883 | |
| Log-pseudolikelihood | -5691.349 | | -6244.257 | | -3713.417 | |
| ***Hypothesis tests*** |  |  |  |  |  |  |
| Clinical efficacy | -11.73 (p<0.001) |  | -7.95 (p<0.001) |  | -9.93 (p<0.001) |  |
| All traumas | 8.42 (p=0.134) |  | 4.43 (p=0.489) |  | 5.80 (p=0.326) |  |
| Other analgesics | 0.53 (p=0.971) |  | 0.41 (p=0.982) |  | 0.61 (p=0.961) |  |
| Serious adverse event | 2.47 (p=0.291) |  | 2.45 (p=0.295) |  | 2.62 (p=0.270) |  |
| ***Time to trough pain*** | 26.44  (0.74; 25.00-27.88) | 44.46  (2.49; 39.57-49.34) | 26.50  (0.75; 25.03-27.97) | 41.77  (1.48; 38.87-44.66) | 26.57  (0.76; 25.08-28.05) | 40.77  (3.11; 34.67-46.87) |

Table S2.4 Pathway estimates

| **Statistic** |  |  |  |  |  |  |
| --- | --- | --- | --- | --- | --- | --- |
| ***Duration severe pain*** | 10.54  (0.70; 9.16-11.91) | Not predicted to exit severe pain state | 10.47  (0.69; 9.11-11.83) | 20.09  (1.12; 17.88-22.29) | 9.66  (0.68; 8.32-11.00) | 37.53  (6.58; 24.64-50.42) |
| ***Time to trough pain*** | 26.94  (0.74; 25.50-28.38) | 44.96  (2.49; 40.07-49.84) | 27.00  (0.75; 25.53-28.47) | 42.77  (1.48; 39.87-45.66) | 27.07  (0.76; 25.58-28.55) | 45.77  (3.11; 39.67-51.87) |
| ***Pain level at trough*** | -1.975  (0.177; 0.000) | -0.819  (0.178; 0.000) | -2.162  (0.197; 0.000) | -1.814  (0.189; 0.000) | -1.891  (0.277; 0.000) | -0.762  (0.287; 0.008) |
| ***Hypothesis tests*** |  |  |  |  |  |  |
| Equality in times to trough | 54.55 (p<0.001) |  | 59.09 (p<0.001) |  | 53.18 (p<0.001) |  |
| Equality in level at trough | 25.79 (p<0.001) |  | 6.61 (p=0.010) |  | 20.62 (p<0.001) |  |
