# Supplementary material for: Evaluation of the effectiveness and costs of inhaled methoxyflurane versus usual analgesia for prehospital injury and trauma: non-randomised clinical study
Source: BMC Emerg Med. 2022 Jul 7;22:122. doi: 10.1186/s12873-022-00664-y (PMC9261021; doi:10.1186/s12873-022-00664-y)
Supplement: Supplementary file 3 — Additional file 3: Supplement3. Statistics. [file 12873_2022_664_MOESM3_ESM.docx]

Supplement 3: Statistics

## Variables

Denote pain due to trauma by $Y^{*}$, where it is an unobservable continuously distributed random variable. Let $S_{ij}=s\in\{0,1,\ldots,10\}$ denote the VNPS score reported by patient $i=1,\ldots,N$ taken in sequence $j=0,1,\ldots,T_{i}$ over time, where $S_{i0}$ denotes the baseline score and $T_{i}$ is the total number of scores taken on patient $i$; note that should the score at the time of baseline not be observed then we imputed its value using the last observation carried forward rule. Scores are collected while the patient is under the influence of the target analgesic but not beyond that time, the target analgesic may be either methoxyflurane or one of its comparators. Table S3.1 gives our assumptions on onset and duration for each analgesic.

**Table S3.1 Onset and duration of analgesia**

| **Analgesic** | **Route** | **Effect begins** | **Effect ends** |
| --- | --- | --- | --- |
| Methoxyflurane | inhaled | +30 secs after administration | +5 mins after discontinuation or +45 mins after administration |
| Entonox® | inhaled | +30 secs after administration | +5 mins after discontinuation or  +45 mins after administration |
| Potency 1 | oral | +30 mins after administration | +6 hrs after administration |
| Potency 2 | oral | +30 mins after administration | +6 hrs after administration |
| Potency 3 | oral | +30 mins after administration | +6 hrs after administration |
| Potency 3 | IV | +1 min after administration | +4 hrs after administration |
| Paracetamol | IV | +5 mins after administration | +6 hrs after administration |

Notes:

Intermittent use is assumed for methoxyflurane and Entonox®

Potency 1 (mild): oral paracetamol or ibuprofen

Potency 2 (moderate): oral paracetamol/mild opiate combination

Potency 3 (high): oral opiate or tramadol, parenteral opiate or paracetamol

The relationship between pain and pain score is established by the following set of (ten) observation rules:

$\Pr\left( S_{ij}=s|\boldsymbol{X} \right)=\Pr\left( \alpha_{s}<Y_{ij}^{*}<\alpha_{s+1}|\boldsymbol{X} \right)$ (1)

where the conditioning variables $\boldsymbol{X}$ contain all the regressors in the statistical model that is to be specified for $Y^{*}$, where we note here that $\boldsymbol{X}$ will include an individual random effect. The cut parameters, collected into vector $\boldsymbol{\alpha}=(\alpha_{0},\alpha_{1},\ldots,\alpha_{11})$, are ordered such that $\alpha_{0}<\alpha_{1}<\ldots<\alpha_{11}$; as we assume $Y^{*}$ to be normally distributed then set fixed are $\alpha_{0}=-\infty$ and $\alpha_{11}=+\infty$. The point in time (measured in minutes) at which the $j^{\mathrm{th}}$ pain score is taken from the $i^{\mathrm{th}}$ patient is denoted $t_{ij}\geq0$, where baseline $t_{i0}=0$ is set fixed and corresponds to when the target analgesic was administered. It is important to note that at baseline the effect on pain of the target analgesic has not yet onset (cf Table S3.1).

## Model

Under analgesia we expect pain to diminish at a decreasing rate over time. Accordingly, we specified quadratic functions of time to represent the pathways of the response of pain to analgesics. The statistical model was specified with the following structure:

$Y_{ij}^{*}=\left( \beta_{0}+\theta_{0}d_{i} \right)+\left( \beta_{1}+\theta_{1}d_{i} \right)t_{ij}+\left( \beta_{2}+\theta_{2}d_{i} \right)t_{ij}^{2}+d_{i}\boldsymbol{\gamma}^{'}\boldsymbol{x}_{it}+(1-d_{i})\boldsymbol{\delta}^{'}\boldsymbol{w}_{it}+u_{i}+\varepsilon_{ij}$ (2)

where the binary dummy $d_{i}$ is defined such that $d_{i}=1$ indicated that methoxyflurane was administered to patient $i$ otherwise when $d_{i}=0$ the comparator was the target analgesic. We assumed the individual effect $u_{i}$ was random (in particular $N(0,\sigma_{u}^{2})$) and independent of all other factors included in the model. Independent regressors were collected into (column) vectors $\boldsymbol{x}$ and $\boldsymbol{w}$, associated parameter coefficients were contained respectively in vectors $\boldsymbol{\gamma}$ and $\boldsymbol{\delta}$ which may vary by target analgesic even if the regressor was common. When regressors were common, we tested for difference of effect using standard single and joint tests. The error term $\varepsilon_{ij}$ was assumed independent and identically $N(0,\sigma_{\varepsilon}^{2})$ for all $(i,j)$ pairs. Departures from this ideal were possible so cluster robust standard errors were computed. Two restrictions were required for parameter identification, set were: $\beta_{0}=0$ and $\sigma_{\varepsilon}^{2}=1$. We used STATA®’s “xtoprobit” routine to estimate the parameters of the model: ($\boldsymbol{\alpha,}\theta_{0},\theta_{1},\theta_{2},\beta_{1},\beta_{2}\boldsymbol{,\gamma,\delta,}\sigma_{u}^{2}).$ Estimates were consistent and asymptotically normally distributed under standard statistical assumptions.

## Main hypothesis test

Our expectations of response under analgesia over the course of time were consistent with parameter $\beta_{1}<0$. Use of methoxyflurane will attenuate pain faster than its comparator provided the difference parameter $\theta_{1}<0$. We therefore conducted the one-sided hypothesis test (Student t statistic; null distribution approximate $N(0,1)$):

$H_{0}: \theta_{1}=0$ versus $H_{1}: \theta_{1}<0$ (3)

where rejection of the null hypothesis $H_{0}$ provides statistically significant evidence that methoxyflurane relieves pain faster than its comparator.

Finally, additional parameters of interest include the time taken to reach minimum pain or “trough pain”. From (2), methoxyflurane’s trough pain is predicted to occur at time (in minutes)

$-\frac{\beta_{1}+\theta_{1}}{2(\beta_{2}+\theta_{2})}$ (4)

and for its comparator at time ${{-\beta}_{1}}/{2\beta_{2}}.$

## Scenario analyses

The following scenario extracts from model (2) a function of time that represents the impact of analgesia on pain over time - the so-called ‘pain pathway’ - that we compared across treatments. For an assigned value of baseline pain, denoted $\omega_{0}$, and incorporating into this the duration until onset of analgesia, denoted $L_{d}$, pain pathways are given by:

$P_{d}\left( t \right)=\omega_{0}+I\left\{ {t\geq L}_{d} \right\}\left[ \left( \beta_{1}+\theta_{1}d \right)(t-L_{d})+(\beta_{2}+\theta_{2}d)({t-L_{d})}^{2} \right]$ (5)

where indicator $I\left\{ A \right\}=1$ if event $A$ is true, otherwise 0. Both pathways, methoxyflurane $P_{1}\left( t \right)$ and comparator $P_{0}\left( t \right)$, had two segments separated according to the length of time in minutes from administration until the onset of analgesia: $L_{1}=0.5$ for methoxyflurane, and comparator $L_{0}$ were given in Table S3.1. The duration that an analgesic takes to reduce pain from level $\omega_{0}$ to a given level $\omega_{1}<\omega_{0}$ is the solution for $t$ to the quadratic equation $P_{d}\left( t \right)=\omega_{1}$.

We used (5) to estimate the durations a patient spent in severe pain under methoxyflurane and comparator. In particular, we set $\omega_{0}=\alpha_{10}$, the level equivalent to the cusp between pain scores of 9 and 10, and $\omega_{0}=\alpha_{7}$, equivalent to the cusp between pain scores of 6 and 7. We then constructed the predicted pathways, $\hat{P}_{d}\left( t \right)$, where all unknown parameters in (5) were replaced with model estimates and solved for durations in $\hat{P}_{d}\left( t \right)=\hat{\alpha}_{7}$. Graphs of predicted pain pathways $\hat{P}_{d}\left( t \right)$ circumscribed by 95% confidence intervals are provided.
